# The TNFR1 antagonist Atrosimab reduces neuronal loss, glial activation and memory deficits in an acute mouse model of neurodegeneration

**DOI:** 10.1038/s41598-023-36846-2

**Published:** 2023-06-30

**Authors:** Natalia Ortí-Casañ, Ate S. Boerema, Karina Köpke, Amber Ebskamp, Jan Keijser, Yuequ Zhang, Tingting Chen, Amalia M. Dolga, Kerensa Broersen, Roman Fischer, Klaus Pfizenmaier, Roland E. Kontermann, Ulrich L. M. Eisel

**Affiliations:** 1 Department of Molecular Neurobiology, Groningen Institute for Evolutionary Life Sciences, University of Groningen, Groningen, The Netherlands; 2 Applied Research Center, Van Hall Larenstein University of Applied Science, Leeuwarden, The Netherlands; 3 Applied Stem Cell Technology, Faculty of Science and Technology, University of Twente, Enschede, The Netherlands; 4 Stuttgart Research Center Systems Biology, University of Stuttgart, Stuttgart, Germany; 5 Institute of Cell Biology and Immunology, University of Stuttgart, Stuttgart, Germany; 6 Department of Molecular Pharmacology, Groningen Research Institute of Pharmacy, University of Groningen, Groningen, The Netherlands

**Keywords:** Tumour-necrosis factors, Neurological disorders, Pharmaceutics, Neuroimmunology

## Abstract

Tumor necrosis factor alpha (TNF-α) and its key role in modulating immune responses has been widely recognized as a therapeutic target for inflammatory and neurodegenerative diseases. Even though inhibition of TNF-α is beneficial for the treatment of certain inflammatory diseases, total neutralization of TNF-α largely failed in the treatment of neurodegenerative diseases. TNF-α exerts distinct functions depending on interaction with its two TNF receptors, whereby TNF receptor 1 (TNFR1) is associated with neuroinflammation and apoptosis and TNF receptor 2 (TNFR2) with neuroprotection and immune regulation. Here, we investigated the effect of administering the TNFR1-specific antagonist Atrosimab, as strategy to block TNFR1 signaling while maintaining TNFR2 signaling unaltered, in an acute mouse model for neurodegeneration. In this model, a NMDA-induced lesion that mimics various hallmarks of neurodegenerative diseases, such as memory loss and cell death, was created in the nucleus basalis magnocellularis and Atrosimab or control protein was administered centrally. We showed that Atrosimab attenuated cognitive impairments and reduced neuroinflammation and neuronal cell death. Our results demonstrate that Atrosimab is effective in ameliorating disease symptoms in an acute neurodegenerative mouse model. Altogether, our study indicates that Atrosimab may be a promising candidate for the development of a therapeutic strategy for the treatment of neurodegenerative diseases.

## Introduction

Tumor necrosis factor alpha (TNF-α) is a master pro-inflammatory cytokine secreted by activated immune cells upon infections or tissue damage. TNF-α plays a key role in the initiation of the innate and adaptive immune system and its expression is thus tightly regulated^1^. However, alterations in the levels of TNF-α can be detrimental and have been related to several autoimmune and neurodegenerative diseases. Indeed, increased levels of TNF-α have been reported in cases of Alzheimer’s disease (AD), multiple sclerosis (MS) or rheumatoid arthritis (RA), among others^2,3^. Blocking of TNF-α that counteracts its elevated levels is successfully used for the treatment of different autoimmune disorders^4^ but largely failed as therapeutic for neurological diseases^5–8^. The failure of anti-TNF therapeutics in neurological diseases might be explained by the opposing functions of the two TNF receptors: stimulation of TNF receptor 1 (TNFR1) activates pro-inflammatory and apoptotic pathways whereas stimulation of TNF receptor 2 (TNFR2) promotes tissue regeneration and neuroprotective pathways^9,10^. TNFR1 is ubiquitously expressed in all cell types while TNFR2 is expressed at low levels in immune cells, endothelial cells and cells of the central nervous system (CNS), such as neurons, microglia, astrocytes or oligodendrocytes^11–14^.

The elevated TNF-α levels observed in neurodegenerative diseases have also been linked to the dysregulation of glutamate metabolism, a key excitatory neurotransmitter in the CNS. Increased TNF-α levels have shown to inhibit the ability of astrocytes to clear glutamate from the extracellular space, resulting in the accumulation of glutamate and consequent overactivation of the glutamate receptors *N*-methyl-d-aspartate (NMDA) and *α*-amino-3-hydroxy-5-methyl-4-isoxazolepropionic acid (AMPA)^15^. This overactivation of glutamate receptors may lead to glutamate-induced excitotoxicity and progressive neuronal cell death as well as learning and memory impairments, common hallmarks of neurodegenerative diseases^16^. As a consequence, NMDA receptor antagonists were developed as treatment strategy but were unsuccessful in clinical trials^17^.

Considering that TNFR1 mediates pro-inflammatory and apoptotic functions and that activation of TNFR2 has been shown to protect neurons against glutamate-induced excitotoxicity^18,19^, an alternative therapeutic approach to treat excitotoxic brain damage could be to selectively block TNFR1 to leave TNFR2 signaling unaffected. Indeed, blocking of TNFR1-mediated signaling led to a reduction of pathological symptoms in the experimental autoimmune encephalitis (EAE) mouse model of MS^20^. Moreover, absence of TNFR1 led to a strong reduction of neurodegeneration in a retinal ischemia mouse model while TNFR2 depletion significantly increased neurodegeneration in the same model^21^.

Further, TNFR1 specific antagonistic antibody Atrosab protected cholinergic neurons from glutamate-induced excitotoxicity and rescued memory deficits in a mouse model of NMDA-induced acute neurodegeneration^22^. Even though Atrosab indicated therapeutic efficacy in animal models^22,23^, it also showed dose-limiting side effects in a clinical phase 1 study, presumably due to bivalent TNFR1 binding and receptor activation^24^. In support of this, Atrosab showed marginal TNFR1 agonistic activity in vitro^24^. Hence, a monovalent derivative of Atrosab, Atrosimab, was developed by genetic engineering. Atrosimab is devoid of any TNFR1 agonistic activity and retains its high TNFR1 selective antagonistic activity in in vitro models and has shown promising effects in mouse models of acute and chronic inflammation^25–27^.

In the present study, we investigated the TNFR1 blocking activity of Atrosimab in an in vivo mouse model of acute cholinergic neurodegeneration and memory impairments through stereotactic injections of NMDA into the nucleus basalis magnocellularis (NBM). The NBM is a cell cluster in the basal forebrain containing cholinergic neurons projecting into the cortex of humans and mice^28^. Overstimulation of glutamate receptors results in cholinergic neuron excitotoxicity, subsequent loss of cholinergic fibers and projections, as well as increased glial activity around the injection site and cognitive deficits^29^. This animal model therefore reflects important aspects of disease pathology at early stages of neurodegenerative diseases, such as AD. This study aims to validate the in vivo therapeutic activity of the novel TNFR1 antagonist Atrosimab in this mouse model of acute neurodegeneration.

## Results

### Atrosimab improves NMDA induced memory impairment

**Figure 1:**
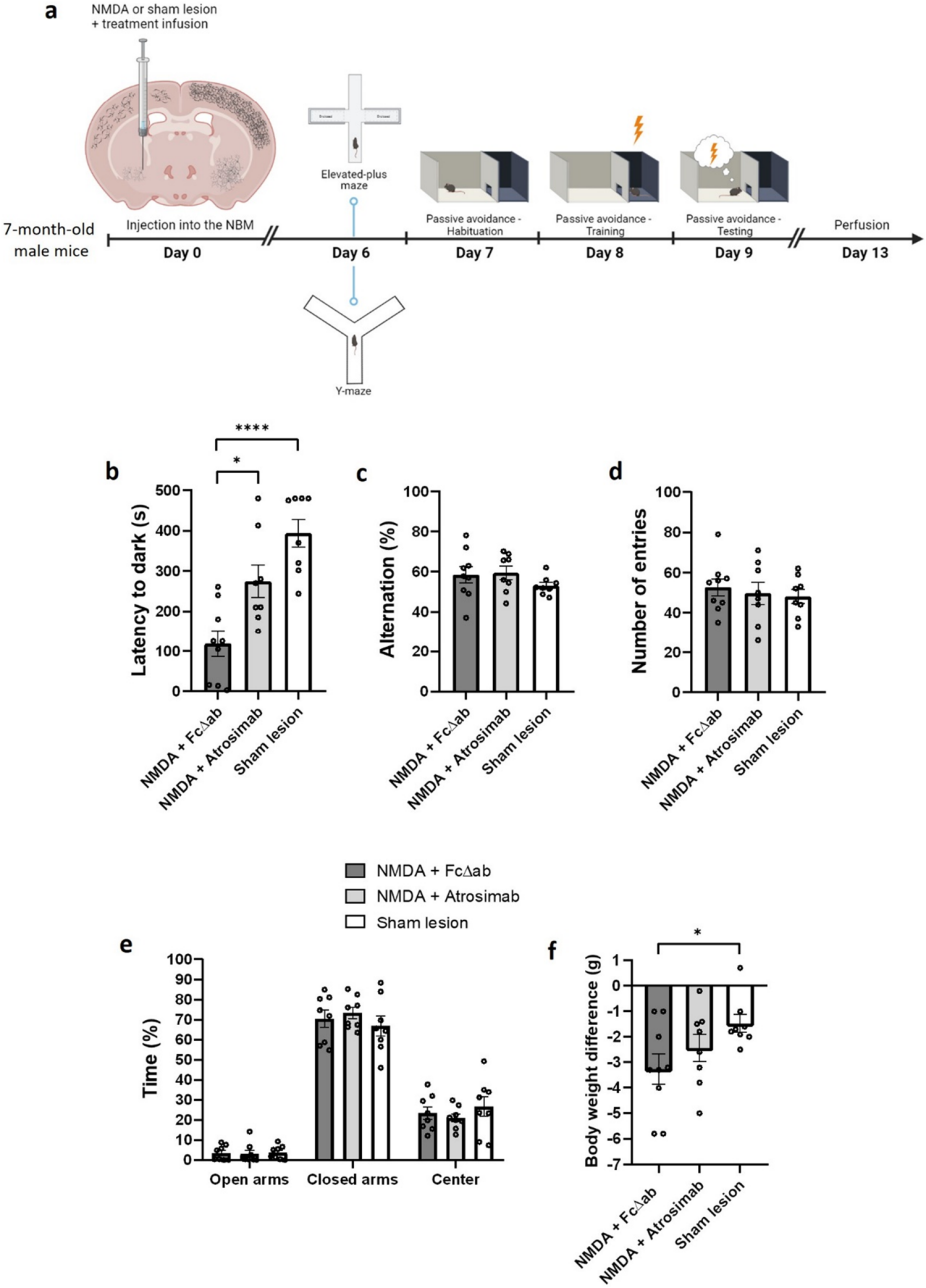
Treatment with Atrosimab rescues NMDA-induced memory deficits. (**a**) Timeline of experimental procedures. (**b**) Post-shock latencies were measured in the passive avoidance paradigm. (**c**) Percentage of alternations and (**d**) number of total entries measured in the spontaneous alternation Y-maze. (**e**) Percentage of time spent in open arms, closed arms and center in the EPM test. (**f**) Body weight differences throughout the experiment (NMDA + FcΔab n = 9; NMDA + Atrosimab n = 8; Sham lesion n = 8; one-way ANOVA, Tukey post hoc analysis). Data are presented as mean ± SEM. *p < 0.05; ****p < 0.0001.

Upon NBM lesion, different behavioral tests were performed to measure cognitive functions (Fig. 1a). To test the effect of Atrosimab treatment on long-term associative memory performance, mice were subjected to the passive avoidance paradigm. After receiving the electric shock, post-shock latencies were significantly longer than pre-shock latencies in all experimental groups (F_1,44_ = 137.5; DF = 1; *p* < 0.001) (Supplementary Fig. S1a). In NBM lesioned animals, Atrosimab treatment (NMDA + Atrosimab) showed no differences in pre-shock latency compared to NMDA + treatment with control protein (NMDA + FcΔab) or in the sham lesioned control group (Supplementary Fig. S1b), indicating a functional test paradigm and no interference of Atrosimab with the sensitivity of the test itself.

NMDA + FcΔab co-injection into the NBM resulted in significantly shorter post-shock latencies (118.8 ± 32.03 s) compared to sham lesion controls (393.9 ± 34.27 s; F_2,22_ = 15.32; DF = 2; *p* < 0.0001), indicating that memory is affected by the NMDA lesion. Co-injection with Atrosimab counteracted this effect, as the post-shock latency was significantly increased (274.8 ± 40.82 s) compared to co-injection with FcΔab (*p* < 0.05) (Fig. 1b), showing increased memory performance after Atrosimab treatment.

To assess short-term memory, anxiety-like behaviour and locomotor activity, mice were tested in the spontaneous alternation Y-maze and elevated-plus maze (EPM). No significant differences were observed in the percentage of alternations (NMDA + FcΔab = 58.59 ± 4.09%; NMDA + Atrosimab = 59.48 ± 3.39%; Sham lesion = 53.04 ± 1.63%) nor the number of total entries (NMDA + FcΔab = 52.66 ± 4.24; NMDA + Atrosimab = 49.62 ± 5.50; Sham lesion = 48.12 ± 3.60) performed in the spontaneous alternation Y-maze test across groups (Fig. 1c,d). Additionally, there were no significant differences in the percentage of time spent in the center (NMDA + FcΔab = 23.36 ± 3.16%; NMDA + Atrosimab = 21.06 ± 1.97%; Sham lesion = 26.62 ± 4.92%), open (NMDA + FcΔab = 3.49 ± 1.29%; NMDA + Atrosimab = 3.15 ± 1.75%; Sham lesion = 3.78 ± 1.17%) or closed arms (NMDA + FcΔab = 70.44 ± 4.24%; NMDA + Atrosimab = 73.29 ± 2.85%; Sham lesion = 66.83 ± 4.91%) in the EPM test (Fig. 1e). Similarly, no changes in the total distanced moved in the EPM were observed, indicating that locomotor activity was not affected by NMDA injections or Atrosimab (Supplementary Fig. S1c). Finally, changes in body weight from day 0 (surgery) until day 13 (perfusion) were evaluated. Co-injection of NMDA + Atrosimab treatment did not influence body weight, since no significant differences were observed compared to sham lesioned mice (NMDA + Atrosimab = − 2.43 ± 0.54 g; Sham lesion = − 1.47 ± 0.34 g) (Fig. 1f). However, injection of NMDA in absence of Atrosimab caused a significant reduction in body weight compared to the sham lesion group (NMDA + FcΔab = − 3.26 ± 0.59 g; Sham lesion = − 1.47 ± 0.34 g; F_2,22_ = 3.09; DF = 2; *p* < 0.05) (Fig. 1f).

### Atrosimab reduces NMDA-induced cholinergic fiber loss in the somatosensory cortex

**Figure 2:**
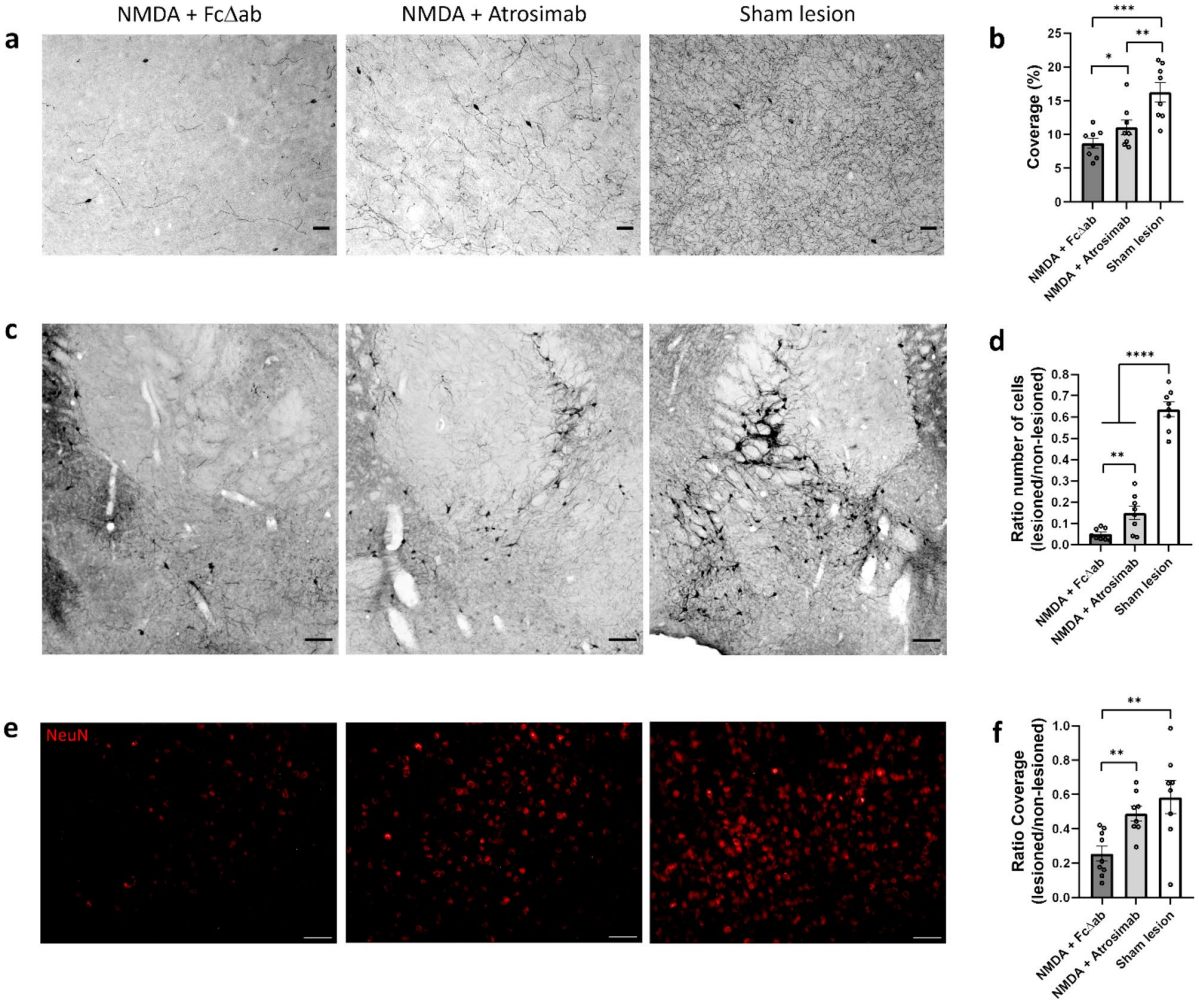
NMDA-induced loss of cholinergic fibers is prevented with Atrosimab treatment. (**a**) ChAT-positive neuronal fiber density measured in layer V of the somatosensory cortex. Representative images of cortical layer V are shown. Scale bar, 50 µm. (**b**) Quantification of area fraction covered by ChAT-positive cholinergic fibers in (**a**) (NMDA + FcΔab n = 8; NMDA + Atrosimab n = 8; Sham lesion n = 8; one-way ANOVA, Tukey post hoc analysis). (**c**) ChAT-positive cholinergic neurons measured in the NBM. Representative images of the NBM (coordinates respective to Bregma: − 0.60 mm) are shown. Scale bar, 100 µm. (**d**) Quantification of the ratio between the number of cholinergic neurons in the lesioned vs. non-lesioned hemisphere of the NBM in (**c**) (NMDA + FcΔab n = 8; NMDA + Atrosimab n = 8; Sham lesion n = 8; one-way ANOVA, Tukey post hoc analysis). (**e**) NeuN-positive neurons measured in layer V of the somatosensory cortex. Representative images of cortical layer V are shown. Scale bar, 50 µm. (**f**) Quantification of the ratio between the fraction of the surface covered by NeuN-positive neurons in the lesioned vs. non-lesioned hemisphere in (**e**) (NMDA + FcΔab n = 9; NMDA + Atrosimab n = 8; Sham lesion n = 8; one-way ANOVA, Tukey post hoc analysis). Data are presented as mean ± SEM. *p < 0.05; **p < 0.01; ***p < 0.001; ****p < 0.0001.

To investigate whether Atrosimab can counteract NMDA-induced cholinergic fiber loss, cholinergic fiber densities in the cortex were assessed. Surface area of choline acetyl transferase (ChAT)-positive fibers in layer V of the somatosensory cortex (coordinates respective to Bregma: − 0.6 to − 1 mm) was measured, since this cortical area mainly receives the cholinergic projections from the NBM^30^. NMDA injection into the NBM led to a significant reduction of ChAT-positive fibers in layer V of the cortex (8.69 ± 0.74%) compared to Sham lesioned mice (16.25 ± 1.43%; F_2,21 _= 11.84; DF = 2; *p *< 0.001) (Fig. 2a). Co-injection of NMDA + Atrosimab reduced the NMDA-induced cholinergic fiber loss, as Atrosimab co-injection significantly increased the number of ChAT-positive fibers (11.04 ± 1.08%) compared to co-injection of FcΔab (*p *< 0.05) (Fig. 2a,b).

To further assess the effect of Atrosimab co-injection on the cholinergic system after NMDA injection, the ratio between the number of cholinergic neurons in the lesioned and the non-lesioned hemisphere of the NBM was calculated. Injection of NMDA into the NBM significantly reduced the number of cholinergic neurons (0.04 ± 0.009) compared to Sham lesioned mice (0.63 ± 0.033; F_2,21 _= 136.4; DF = 2; *p *< 0.0001) (Fig. 2c). However, this effect was attenuated by co-injection of NMDA + Atrosimab, which led to a significant increase in the number of cholinergic neurons in the NBM (0.14 ± 0.031) compared to NMDA + FcΔab co-injection (*p* < 0.01). Besides, Sham lesioned mice showed significantly higher number of cholinergic neurons than both NMDA co-injection groups (*p *< 0.0001) (Fig. 2c,d).

We also investigated whether Atrosimab was able to protect neurons in general. To this end, we measured NeuN as pan-neuronal marker in layer V of the somatosensory cortex^31^. The ratio between the surface area of NeuN positive nuclei in the lesioned and the non-lesioned hemisphere in layer V of the somatosensory cortex was calculated. NMDA injection into the NBM resulted in a significant decrease of this ratio (0.25 ± 0.04) compared to the Sham group (0.58 ± 0.09; F_2,22 _= 7.22; DF = 2; *p* < 0.01) (Fig. 2e). This decrease was prevented by co-injection of NMDA + Atrosimab, shown by a significant increase in the ratio of NeuN-positive surface area (0.48 ± 0.04) compared to NMDA + FcΔab administration (*p *< 0.01) (Fig. 2e,f). 

To investigate if Atrosimab could affect neuronal firing, we recorded the spontaneous activity of cultured neurons in 48-well plates before and 24h following Atrosimab treatment. We observed no significant differences in the mean firing rate in primary neurons with (0.54 ± 0.10) and without Atrosimab (0.60 ± 0.06; t = 0.56; DF = 7; *p *= 0.58) (Supplementary Fig. S2). Next, we applied glutamate to primary neurons to increase neuronal excitability and investigate whether Atrosimab was able to alter the increased neuronal excitability. The analysis of mean firing rates in neurons challenged with glutamate (0.71 ± 0.06) showed a significant increase in the firing rate compared to initial neuronal firing activity (t = 3.52; DF = 7; *p *< 0.01) (Supplementary Fig. S2). However, Atrosimab pre-treatment (0.82 ± 0.10) did not reduce glutamate-induced increased neuronal firing activity (t = 0.81; DF = 7; *p *= 0.44) (Supplementary Fig. S2).

Finally, to more specifically investigate the effect of Atrosimab on neuronal synaptic plasticity, we assessed the expression levels of synapsin-1, a marker related to neurotransmitter release, in the hippocampus^32^. The hippocampus was chosen as candidate area due to being a plastic brain region, useful for assessing a plasticity marker. Moreover, the hippocampus is also innervated by cholinergic projections from the NBM^33^. We observed that synapsin-1 levels remained unaltered across groups (Supplementary Fig. S3a,b), indicating that neither NMDA (0.79 ± 0.04) nor Atrosimab (0.81 ± 0.05) co-injections influenced neurotransmitter release in our model (F_2,21 _= 0.09; DF = 2; *p *= 0.91).

### NMDA-induced gliosis in the NBM is reduced after Atrosimab treatment

**Figure 3:**
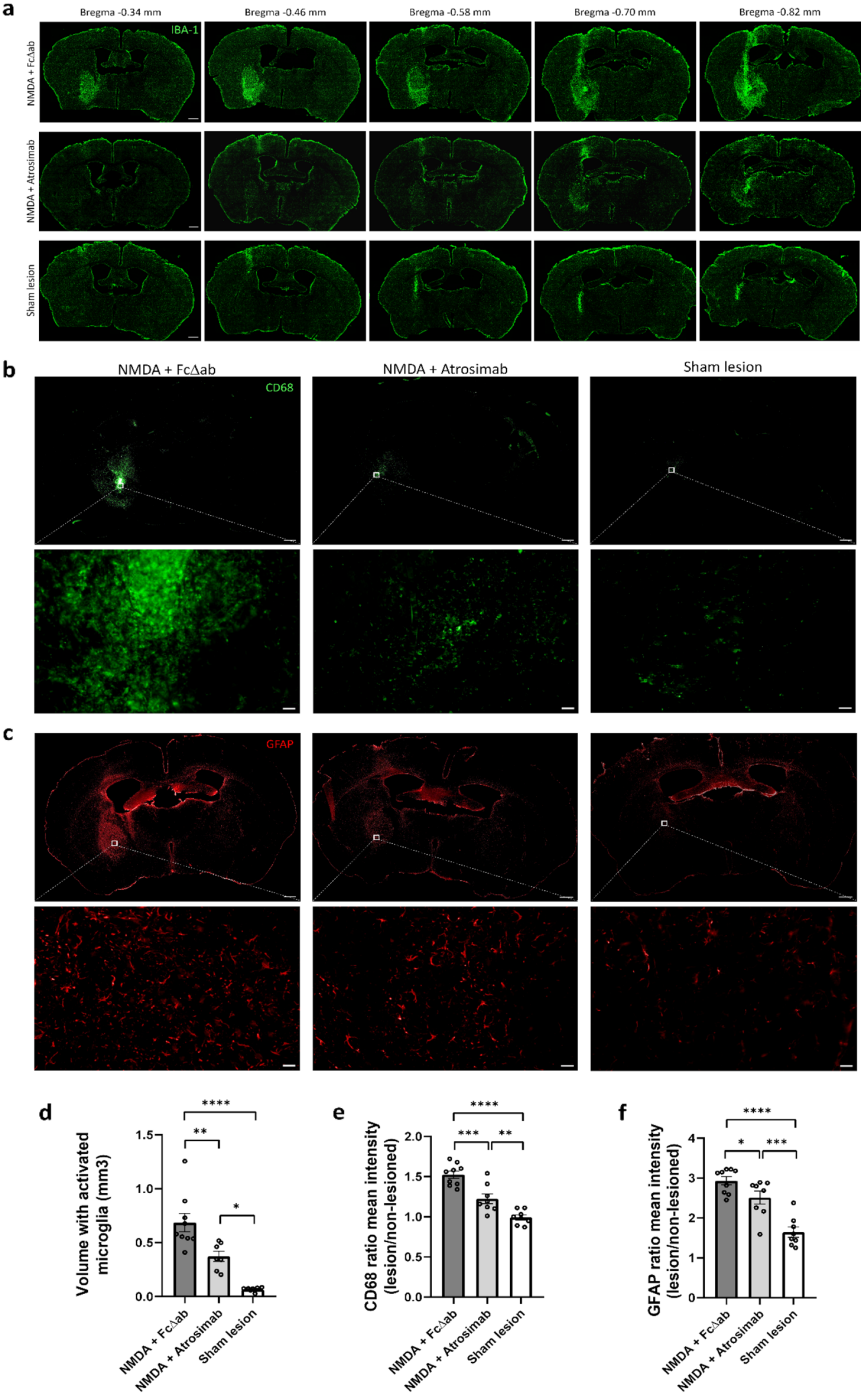
Atrosimab reduces NMDA-induced gliosis area at the lesion site. (**a**) Progression of NMDA-induced lesion in the NBM. Sections were stained with IBA-1. Brighter signal indicates more activated microglia. Representative images of the NBM (coordinates respective to Bregma: − 0.34 to − 0.82) are shown. Scale bar, 200 µm. (**b**) CD68-positive microglia measured in the NBM. Representative images of the NBM (coordinates respective to Bregma: − 0.60 mm) are shown. Scale bar, 200 µm. (**c**) GFAP-positive astrocytes measured in the NBM. Representative images of the NBM (coordinates respective to Bregma: − 0.60 mm) are shown. Scale bar, 200 µm. (**d**) Quantification of the volume of IBA-1-positive microglia in (**a**) (NMDA + FcΔab n = 9; NMDA + Atrosimab n = 7; Sham lesion n = 8; one-way ANOVA, Tukey post hoc analysis). (**e**) Quantification of the ratio between the mean intensity of the region of interest covered by CD68-positive microglia in the lesioned vs. non-lesioned hemisphere in (**b**) (NMDA + FcΔab n = 9; NMDA + Atrosimab n = 8; Sham lesion n = 8; one-way ANOVA, Tukey post hoc analysis). (**f**) Quantification of the ratio between the mean intensity of the region of interest covered by GFAP-positive astrocytes in the lesioned vs. non-lesioned hemisphere in (**c**) (NMDA + FcΔab n = 9; NMDA + Atrosimab n = 8; Sham lesion n = 8; one-way ANOVA, Tukey post hoc analysis). Data are presented as mean ± SEM. *p < 0.05; **p < 0.01; ***p < 0.001; ****p < 0.0001.

Lesions in the NBM were generated by stereotactic injections of NMDA. To determine lesion size and gliosis, microglial activation was evaluated at the height of the NBM. Injection of NMDA + FcΔab resulted in a significantly larger lesion (0.686 mm^3^ ± 0.085) throughout the NBM (coordinates respective to Bregma: − 0.34 to − 0.82) compared to sham lesioned animals (0.067 mm^3^ ± 0.007; F_2,20_ = 24.76; DF = 2; *p* < 0.0001) (Fig. 2a). Co-injection of NMDA + Atrosimab showed a protective effect, as gliosis as well as lesion size was significantly reduced to approximately half the size (0.373 mm^3^ ± 0.047) of the NMDA + FcΔab co-injection group (*p* = 0.0054) (Fig. 3a,d). Gliosis and lesion size in the sham lesioned animals were still significantly smaller than in the Atrosimab animals (*p* = 0.0103) (Fig. 3a,d).

To further evaluate the effect of Atrosimab on microglial phenotypes, we investigated changes in CD68, a specific marker found in lysosomes of microglia and related to microglial phagocytosis^34^. We found that injection of NMDA + FcΔab led to a significant increase in the ratio between the mean intensity of CD68-positive microglia in the lesioned and the non-lesioned NBM hemisphere (1.52 ± 0.044) compared to control animals (0.99 ± 0.031; F_2,22_ = 32.18; DF = 2; *p* < 0.0001) (Fig. 3b). NMDA + Atrosimab co-injection strongly attenuated CD68-positive microglial responses, as the ratio in this group (1.22 ± 0.06) was significantly reduced compared to NMDA + FcΔab co-injection (*p* < 0.001). Moreover, CD68-positive ratio in the NMDA + Atrosimab group was still significantly higher than sham lesioned animals (*p* < 0.01) (Fig. 3b,e).

Astrocytes are a type of glial cell present in the CNS, which play an important role in glutamate homeostasis and in mediating inflammatory responses. Thus, we investigated whether Atrosimab influenced astrocytic activation by measuring the ratio between the mean intensity of GFAP-positive cells, a specific marker for activated astrocytes, in the lesioned and the non-lesioned NBM hemisphere. Injection of NMDA into the NBM resulted in a significant increase in this ratio in and around the NMDA-induced lesion (2.93 ± 0.10) compared to sham animals (1.64 ± 0.13; F_2,22_ = 24.31; DF = 2; *p* < 0.0001 (Fig. 3c). However, we observed that co-injection of NMDA + Atrosimab (2.51 ± 0.16) led to a significant reduction in the ratio compared to NMDA + FcΔab co-injection (*p* < 0.05). Nevertheless, the astrocytic activation ratio in sham lesioned animals was significantly lower than both NMDA co-injection groups (*p* < 0.001 and *p* < 0.0001) (Fig. 3c,f).

## Discussion

Even though the role of TNF-α in neurodegeneration has been widely acknowledged, TNF-neutralizing therapies have failed to treat neurological diseases. Previous research has demonstrated the antithetic functions of the two TNF receptors, being TNFR1 activation related to pro-inflammatory pathways while TNFR2 activation promotes neuroprotection. In fact, different studies have demonstrated the potential of specifically targeting TNFRs in different neurodegenerative models. For instance, the use of a specific bivalent TNFR1 antagonist, Atrosab, was effective in disease models of MS^23^ and acute neurodegeneration^22^. However, Atrosab showed limitations during clinical trials^24^. In the present study we aimed to validate the novel monovalent TNFR1 antagonist Atrosimab^25,26^ in a NMDA-induced acute neurodegeneration mouse model. We demonstrated that blocking of TNFR1 with Atrosimab decreases the area with activated glial cells around the lesion site, attenuates NMDA-induced neurodegeneration and reduces cognitive impairments.

NMDA injections into the NBM led to a clear impairment in long-term memory functions measured in the PA paradigm. Co-injection of Atrosimab showed an improvement in fear-related long-term memory functions compared to co-injection with a control protein. This is combined with a reduction in the loss of cholinergic neurons in the NBM as well as cholinergic fibers in layer V of the somatosensory cortex caused by NMDA injections.

These results demonstrate that Atrosimab rescues long-term memory impairment induced by NMDA-mediated neurotoxicity. NMDA injections into the NBM excessively stimulate glutamate receptors, resulting in a sustained influx of Ca^2+^ in the residing cholinergic neurons in the NBM. The increase in intracellular Ca^2+^ activates apoptotic signaling pathways and leads to excitotoxicity and death of cholinergic neurons^35^. The cholinergic neurons in the NBM are the main source of cholinergic projections to the cortex and amygdala, which are essential for long-term associative memory and passive avoidance response^36,37^. Other studies have investigated memory deficits after inducing excitotoxic brain damage. For instance, NMDA injections into the NBM of C57BL/6J mice resulted in memory impairments that were rescued with lovastatin administration, a statin that mediates neuroprotective functions^38^. Similarly, memory impairments produced by phthalic acid injections into the NBM of Sprague–Dawley rats were prevented after administration of the cholinergic agonist oxotremorine^39^. Finally, our results are in line with the study by Dong et al.^22^, where treatment with the TNFR1 antagonist Atrosab rescued NMDA-induced memory deficits evaluated in the PA paradigm.

Contrary to the impaired long-term associative memory, NMDA injections had no effect on prefrontal cortex dependent short-term spatial memory or anxiety-like behavior of mice, since no significant differences were observed in the spontaneous alternation Y-maze test nor EPM, respectively. Short-term spatial memory is mainly mediated by neurons in the prefrontal cortex, which is not innervated by cholinergic neurons residing in the NBM^37^. NMDA injections further showed no effect on anxiety-like behavior or short-term spatial memory in other studies^22,38,40^. Taken together, our behavioral data indicate that the NBM lesions did not impair prefrontal cortex-dependent learning functions but led to behavioral deficits related to degeneration of cholinergic projections and its memory functions, which were prevented with Atrosimab treatment. Finally, we demonstrated that Atrosimab counteracted the loss in body weight caused by NMDA injections and was well tolerated based on survivability throughout the experiment.

Besides demonstrating the protective effect of Atrosimab on the cholinergic system, we investigated whether the same outcome was observed for general neuronal populations using the NeuN marker. Next to direct effect on cholinergic fibers in layer V of the somatosensory cortex, injection of NMDA into the NBM also led to a significant reduction of neuronal coverage in layer V of the somatosensory cortex compared to control animals. Nevertheless, co-injection of NMDA + Atrosimab counteracted neuronal loss, as we observed a significant increase in the amount of surface covered with NeuN-positive neuronal cells in the Atrosimab group compared to NMDA + FcΔab animals, in this area. Overall, our results suggest that Atrosimab exerts a general protective effect in preventing neuronal loss in layer V of the somatosensory cortex after NMDA-induced excitotoxicity.

Furthermore, we investigated synaptic plasticity, neurotransmitter release and neuronal excitability in our model. Previous studies have reported changes in synaptic plasticity and increases in glutamate production, glutamatergic signaling and NMDA receptors expression after induction of an injury or lesion^41,42^. Interestingly, administration of the TNFR1 antagonist XPro1595 normalized the increased neuronal excitability and NMDA receptor levels in a chronic constriction injury mouse model^42^. Based on these data, we evaluated whether synaptic plasticity and neuronal excitability were altered in our model and the potential effects of Atrosimab. Our results revealed that in the dentate gyrus of the hippocampus, levels of synapsin-1, a protein related to synaptic plasticity and neurotransmitter release, were unchanged across groups, indicating that the NMDA-induced lesion did not affect synaptic plasticity in the hippocampus in our model. Additionally, there was no direct effect of Atrosimab on synaptic plasticity in this area. Moreover, we observed that glutamate over-stimulation of primary cortical neurons led to an increase in neuronal firing rates. However, co-treatment with Atrosimab showed no significant effect, indicating that Atrosimab is not able to alter neuronal excitability in this setting.

Sustained activation of NMDA receptors can also lead to the activation of glial cells such as macrophages/microglia and astrocytes. Microglia are the resident macrophages in the CNS and they play a key role in scavenging the brain and reacting against pathogens or insults. To do so, microglia secrete inflammatory molecules, like cytokines and chemokines^43^. Inflammation mediated by microglia is essential to the acute immune response, however, constant activation and release of pro-inflammatory cytokines can lead to chronic neuroinflammation^44^. Upon NMDA injections, we observed an increase in microglial activation and phagocytosis around the lesion site compared to Sham lesion control animals. Co-injection of NMDA + Atrosimab significantly decreased microglial activation around the lesion compared to co-injection of NMDA + FcΔab. Astrocytes mediate inflammatory responses and are responsible for synapse maintenance and neurotransmitter homeostasis. NMDA injections led to a significant increase in astrocytic activation in and around the lesion site compared to control animals. Co-injection of Atrosimab significantly attenuated this effect, leading to a reduction in astrocytic activation compared to NMDA co-injected with control protein. Taken together, our results indicate that Atrosimab reduced inflammatory responses mediated by glial cells and lesion size caused by NMDA injection. Similar effects of NMDA on glial cell activation were observed in previous studies^45,46^.

As mentioned, sustained activation of NMDA receptors leads to excitotoxicity and cell death. Besides, the role of TNFR1 in apoptosis and neuroinflammation is well-established^21,47–49^. Thus, we suggest that blocking TNFR1 through Atrosimab could impair TNF-related neurodegeneration while leaving TNFR2 signaling unaffected. Blocking of TNFR1 might shift TNF-α signaling towards TNFR2, which exerts neuroprotective and anti-inflammatory functions via the PKB/Akt and NFκB pathways^19,21^. This is supported by the finding that upon TNFR1 blocking, expression of TNFR2 and PKB/Akt signaling is enhanced^38^. Moreover, inhibition of TNFR1 would eliminate the competition of membrane TNF to bind to both TNFRs^50^. Thus, in response to NMDA-induced excitotoxicity, the released TNF-α would exclusively activate TNFR2-mediated neuroprotective pathways in different TNFR2-expressing cell types, which may counteract NMDA-induced neurodegeneration. In fact, a study demonstrated that neuronal TNFR1 mediates microglial inflammation and neurotoxicity while neuronal TNFR2 mediates neuroprotection, especially in an excitotoxic environment^45^. Additionally, ablation of TNFR2 in oligodendrocytes prevented the therapeutic effect of Xpro1595, a soluble TNF inhibitor, in the EAE model, demonstrating that the therapeutic effect of soluble TNF/TNFR1 inhibition is dependent on TNFR2 expression^51^. Similarly, a study by Gao et al.^52^ showed that TNFR2-deficient microglia develop a pro-inflammatory profile, possibly mediated by increased TNF-α binding to TNFR1. Finally, blocking TNFR1 signaling with specific antagonistic antibodies was previously shown to be effective in counteracting glutamate excitotoxicity in studies using the NBM lesion model^19,22^. Importantly, it was shown that neuroprotective effects due to selective inhibition of TNFR1 with Atrosab, the bivalent predecessor of Atrosimab, were dependent on TNFR2 and PKB/Akt signaling activity^22^. Furthermore, blocking of TNFR1 with Atrosab also led to an increase in cholinergic fibers in the cortex in a NMDA-induced excitotoxicity mouse model^22^. These findings consolidate the idea that inhibition of TNFR1 and maintenance of TNFR2 is essential for the treatment of CNS diseases. Collectively, these results suggest that, in our study, inhibition of TNFR1 with Atrosimab rescues cholinergic degeneration and cognitive deficits by shifting TNF-signaling to TNFR2 and activating the PKB/Akt signaling pathway.

In the present study we show that the novel TNFR1 antagonistic antibody Atrosimab has therapeutic efficacy in attenuating neurodegeneration in a NMDA-induced excitotoxicity mouse model. Atrosimab reverted neurodegeneration-associated long-term memory impairment assessed in the passive avoidance paradigm, reduced lesion size and inflammation based on microglial and astrocytic activation and rescued cholinergic neurons and its cortical projections. As Atrosimab is a TNFR1 selective antibody, we can conclude that the antibody shows neuroprotective effects via TNFR1 inhibition in an in vivo model. Atrosimab was used in this proof-of-concept study to validate its therapeutic efficacy, as the bivalent TNFR1 antagonist Atrosab showed agonistic TNFR1 activity and dose-limiting side effects in a clinical phase 1 study^24^. Atrosimab compared to Atrosab has higher TNFR1 affinity in vitro, lacks any TNFR1 agonistic activity^25,26,53^and shows similar therapeutic efficacy in different mouse models of acute and chronic inflammation, such as non-alcoholic steatohepatitis (NASH), experimental arthritis and EAE^23^. This study adds further evidence for TNFR1 being an important player in acute neurodegeneration and a promising therapeutic target in both neuroinflammatory and neurodegenerative diseases. We propose that the therapeutic efficacy of Atrosimab is achieved by specifically blocking TNFR1, inhibiting all the TNFR1-related apoptotic and inflammatory pathways known to be activated upon the NMDA insult. Probably of equal importance, under these conditions of TNFR1 specific intervention, the TNFR2-associated neuroprotective pathways remain intact and contribute to protect from neuronal cell death and memory impairments.

In conclusion, our data provides proof-of-concept that TNFR1 inhibition through Atrosimab is effective in ameliorating neurodegenerative processes induced by glutamate excitotoxicity in a mouse model. Limitations of the data interpretation reside in use of a mouse model. Although glutamate excitotoxicity and degeneration of the cholinergic system are prevalent hallmarks in early stages of many neurodegenerative diseases, such as AD, our model does not fully mimic the disease context. Therefore, the role of Atrosimab in treatment of neurodegenerative diseases warrants further investigations in different neurological and inflammatory disease models.

## Materials and methods

### Materials

Atrosimab and the control scrambled protein (FcΔab) were provided by Baliopharm (Basel, Switzerland) and described previously^27^. Briefly, Atrosimab was produced by Catalent (Catalent Pharma Solutions, Madison, Wisconsin, US) in stably transfected Chinese hamster ovary cells and purified by depth filtration, anion exchange hybrid purifier, protein A affinity, anion exchange, CHT multi modal chromatography and virus inactivation.

### Mice

To be able to assess the effects of the humanized antagonistic antibody Atrosimab at the behavioural and pathological level in an in vivo mouse model of acute neurodegeneration, hu/mTNFR1-knock-in (huTNFR1-k/i) mice on a C57Bl/6J background were used. The huTNFR1-k/i mouse line has been generated and previously described^22^. In brief, the endogenous TNFR1 of these animals has been replaced by a chimeric TNFR1 consisting of the human TNFR1 extracellular domains fused to the mouse TNFR1 transmembrane and intracellular domains, resulting in a humanized transgenic mouse line. Male homozygous huTNFR1-k/i mice at 7 months of age were used for the experiment. All animals had access to food and water ad libitum and were on a 12 h:12 h light/dark cycle. All procedures were carried out in accordance with ARRIVE Guidelines and the relevant guidelines and regulations for animal research. All experiments were approved by the Animal Welfare Body and Ethical Committee for the use of experimental animals of the University of Groningen, and the Dutch Central Committee on Animal Studies under license number AVD1050020197306.

### Nucleus basalis magnocellularis lesion model

A unilateral NBM lesion is enough to impact fear related memory in rodents^22,38,39,54^ and was preferred over a bilateral lesion because it allows for the contralateral hemisphere as internal control in analyses and also reduces discomfort for the animals. Injections into the NBM were performed as previously described^22,54^. Briefly, mice were anesthetized with isoflurane (induction 5%/maintenance 1–2% in 60% O_2_ enriched air). Mouse heads were fixated in a Kopf stereotactic frame (Kopf Instruments model 900) in the flat skull orientation. Next, mice were unilaterally injected in the NBM (coordinates relative to Bregma: − 0.04 Anteroposterior, − 0.21 or 0.21 Lateral, − 0.44 and − 0.46 Dorsoventral (DV). The dorsoventral coordinate was measured relative to the top of the skull. A total volume of 0.6 μl solution was slowly infused (0.1 μl/min) at − 0.44 and − 0.46 DV, 0.3 μl at each depth. After infusion, the fluid was allowed to diffuse for 5 min before the needle was slowly retracted. Mice were carefully monitored until total recovery from anesthesia and in the days following surgery. In the subsequent days mice were subjected to different behavioral tests: spontaneous alternating Y-maze (SAM) and elevated plus maze (EPM) on day 6, the passive avoidance paradigm (PA) on days 7–9 after the NBM lesion. Finally, mice were euthanized on day 13 to collect brain tissue. A detailed description of the timeline is depicted in Fig. 1a.

HuTNFR1-k/i mice (n = 26) were randomly allocated to four different treatment groups using the randbetween function in MS Excel: 1) NMDA lesion with control treatment: co-injection of 55 nmol NMDA + 3 μg FcΔab (scrambled sequence antibody), 2) NMDA lesion with Atrosimab treatment: co-injection of 55 nmol NMDA + 3 μg Atrosimab antibody, 3a) Sham lesion with control treatment: co-injection of PBS + 3 μg FcΔab and 3b) Sham lesion with Atrosimab treatment: co-injection of PBS + 3 μg Atrosimab (Table 1). Experimenters were blinded for the injected solutions. For all analyses, group 3a) PBS + FcΔab and 3b) PBS + Atrosimab were combined in one Sham lesion control group, as the differences measured in different behavioral analyses between these groups were negligible (Supplementary Fig. S1). This effect was also observed in the study by Dong et al.^22^ where control protein did not differ from Atrosab treatment.

**Table 1:** Treatment groups and dosages.

Group	Number of animals	Co-injection
1. NMDA + control treatment	9	55 nmol NMDA + 3 μg FcΔab
2. NMDA + Atrosimab treatment	9 (8)*	55 nmol NMDA + 3 μg Atrosimab
3a. Sham lesion + control treatment	4	Sham (PBS) + 3 μg FcΔab
3b. Sham lesion + Atrosimab treatment	4	Sham (PBS) + 3 μg Atrosimab

*NMDA N*-methyl-d-aspartate, *PBS* phosphate buffered saline.

*One animal from group 2) had to be terminated after performing the surgery.

### Behavioral tests

After the NBM injection, mice were subjected to different behavioral tests to assess their cognitive functions.

All behavioral tests were conducted by a researcher blinded for treatment groups. The tests were performed in the subsequent order: Spontaneous alternation Y-maze (day 6), Elevated-plus maze (EPM) (day 6) and Passive avoidance paradigm (PA) (day 7–9). On day 13, mice were terminated and brain tissue was collected.

#### Habituation

To minimize the effects of stress and novelty in the behavioral paradigms, mice were habituated individually to handling by the researcher for 2 min/day in the experimental room starting five days prior to the behavioral tests.

#### Spontaneous alternation Y-maze

Spontaneous alternation was assessed in the Y-maze paradigm to evaluate short-term spatial memory^55^. It serves as a control for unintended cognitive side effects. It is primarily hippocampus dependent^56,57^ and was unaffected in previous NBM lesion studies^22,38^. The Y-maze consisted of three arms (40 cm length × 8 cm width) separated at a 120° angle from each other. Light intensity was adjusted to 10 lx and mice were individually placed in the center of the maze, where they were allowed to freely explore for 10 min. Consecutive entry into all three different arms was defined as a triad. The percentage of spontaneous alternations was manually calculated as follows: number of triads/(total number of entries − 2) × 100.

#### Elevated-plus maze (EPM)

Anxiety-like behavior was assessed in an EPM^58^. The maze was elevated 60 cm above the ground and consisted of two open arms (5.5 cm width × 30 cm length) and two enclosed arms (5.5 cm width × 30 cm length × 15 cm height) situated in a plus-shape. Light intensity was adjusted to 10 lx in the center of the maze and 12 lx in the open arms. Mice were placed in the center of the maze and allowed to freely explore all arms for 8 min. Percentage of time spent in center, open and closed arms as well as number of arm entries, total number of entries and the distance moved were recorded and scored using EthoVision 11.5 software (Noldus, Wageningen, the Netherlands).

#### Passive avoidance (PA) paradigm

Long-term associative memory was assessed with the PA paradigm^22^. The PA paradigm was selected because after damaging the NBM, one of the most prominent behavioural deficits is loss of passive avoidance response, due to damaged cholinergic innervation in the cortex^36^. The apparatus consists of a light (700 lx) and a dark (1–4 lx) compartment separated by a retractable door. On day 7 after NBM injection, mice were habituated to the apparatus by placing them on the light compartment facing the retractable door. After 60 s, the door was lifted and mice were allowed to freely explore both compartments for 5 min. On day 8 after the NBM injection, mice were placed in the light compartment and, after 60 s, the door was lifted and the latency to enter the dark compartment was recorded (pre-shock latency). When mice entered the dark compartment, the retractable door was closed, and mice received an electric shock (0.7 mA, 2 s). Mice were removed from the dark compartment after 30 s, to allow for association of the shock with the context. On day 9 after the NBM injection, memory retention was determined by measuring the latency of the animals to enter the dark compartment (post-shock latency) for maximally 8 min. Hesitance to enter the dark compartment, expressed by long latencies in this test, indicate functional memory of previous shock experience. Short latencies indicate damaged memory.

### Transcardial perfusion and tissue processing

Mice were sacrificed on day 13 after NBM injections. Mice were terminally anesthetised by intraperitoneal (IP) injection of 20% sodium pentobarbital and subsequently transcardially perfused with 0.9% heparin saline to remove the blood, immediately followed by 4% paraformaldehyde (PFA) in PBS (0.1 M, pH 7.40). Brains were collected and post-fixated for 24 h with 4% PFA in PBS and subsequently cryopreserved and dehydrated for at least 24 h using sucrose solution (30% sucrose in PBS). Next, brains were frozen and cut into 20 μm-thick sections using a cryostat and stored at 4 °C in PBS containing 0.1% sodium azide.

### Immunohistochemistry for detection of cholinergic fibers

Free-floating sections were rinsed extensively using PBS (0.01 M, pH 7.40) and subsequently incubated with 0.3% H_2_O_2_ in PBS for 30 min. Next, sections were rinsed with 0.1% Triton X-100 (TX-100) in PBS for 20 min and preincubated in PBS containing 5% normal rabbit serum (NRS) and 0.2% TX-100 at room temperature for 90 min. Thereafter, sections were incubated with primary antibody goat anti-ChAT (1:1000, Lot: 3305977, EMD Millipore Corporation, AB144P-1ML) in PBS containing 5% NRS and 0.2% TX-100 at 37 °C in a water bath for 3 h and subsequently at 4 °C in a cold room for 3 days. After primary antibody incubation, sections were extensively rinsed with 0.1% TX-100 in PBS and incubated with biotinylated rabbit-anti-goat secondary antibody (1:400, Lot: 136139, Invitrogen, A10518) in PBS containing 5% NRS and 0.2% TX-100 at room temperature for 3 h. Then, sections were rinsed with 0.1% TX-100 in PBS and incubated in PBS with Vectastain ABC kit PK-6100 standard (A and B Solution were diluted to 1:500) at room temperature for 2 h. Sections were rinsed with PBS and stored at 4 °C overnight. Cholinergic fibers were then visualised using DAB (Sigma). The contrast was enhanced by using ammonium nickel sulfate. Finally, sections were mounted on glass slides using 1% gelatine solution, air-dried, dehydrated and cover-slipped with DPX as mounting medium. Images were obtained using a Leica DMI6000 B microscope (Leica Microsystems) at a 200× magnification.

### Immunohistochemistry for detection of Iba1, CD68, GFAP, synapsin-1 and NeuN

Free-floating sections were rinsed extensively using tris-buffered saline (TBS) (0.01 M, pH 7.40). Next, sections were preincubated in TBS containing 0.1% TX-100 and 3% bovine serum albumin (BSA) at room temperature for 30 min. Subsequently, sections were incubated with primary antibodies in TBS containing 0.1% TX-100 and 3% BSA at 4 °C for 24 h. After primary antibody incubation and extensive rinsing with TBS, sections were incubated with corresponding Alexa Fluor-conjugated secondary antibodies (AF488-conjugated donkey-anti-rabbit, 1:500, [A21206], AF555-conjugated donkey anti-mouse, 1:500, [A32773], AF488-conjugated donkey anti-rat, 1:500 [A21208]; Invitrogen) in TBS containing 0.1% TX-100 and 3% BSA at room temperature for 2 h. Then, sections were rinsed in TBS again and mounted using Mowiol (Sigma) to prevent fluorescent staining from fading. The following primary antibodies were used: rabbit anti-IBA1 (1:2500, 019-19741, Wako), rat anti-CD68 (1:1000, MCA1957GA, Bio-Rad), mouse anti-GFAP (1:10.000, G3893, Sigma), rabbit anti-synapsin-1 (1:500, AB1543, Millipore) and mouse anti-NeuN (1:500, MAB377, Millipore). Fluorescent images were obtained using a Leica DMI6000 B microscope (Leica Microsystems) at a 100x (CD68, GFAP) or 200x (Iba1, synapsin-1, NeuN) magnification with 10% overlap between tiles. Individual tiles were stitched together by the Leica LAS-X microscope software.

### Microelectrode array (MEA)

Primary cortical neurons were obtained from brain embryos of huTNFR1-k/i mice at embryonic day 14. Meninges and olfactory bulbs were carefully detached from the brains, and cortical neurons were separated by mechanical dissociation. Primary neurons were seeded in medium containing 10 µg/ml of Laminin (Sigma, USA, #3400-010-02) followed by addition of Neurobasal medium. MEA recordings were performed using a Maestro multi-well MEA recorder (Axion Biosystems, USA). Dissociated primary cortical neurons (120 K cells/cm^2^ per each well) were grown in 48-well MEA plate (Axion Biosystems, M768-tMEA-48W) coated with 0.5w/v% polyethyleneimine. Spike detection and neuronal activity was measured before and after compound’s treatment for 24 h on day in vitro 14 and 15 respectively. Axion AxIS Software recorded neuronal spikes for rate analysis.

### Analysis

#### IBA-1, CD68 and GFAP immunohistochemistry

Gliosis around the injection site, at the anterior posterior range of the NBM (coordinates respective to Bregma: from − 0.34 to − 1.06 mm), was assessed by measuring IBA-1 positive volume in serial sections, on average 120 µm apart. The total lesion volume was calculated by linear interpolation between measured sections. For CD68 and GFAP, the mean intensity signal between the lesioned and non-lesioned (control) hemisphere was calculated. For each section, an area of interest was drawn around the fluorescent IBA-1, CD68 or GFAP area based on signal intensity (see Fig. 2) using ICY bioimage analysis software^59^. One mouse from group 2) NMDA + Atrosimab had to be excluded from the IBA-1 analysis due to a high background in the immunohistochemical staining preventing correct analysis. For the IBA-1 immunohistochemistry, we aimed at determining total lesion volume and, thus, 7 serial sections per brain were selected. For CD68 and GFAP, we investigated specific inflammatory responses after Atrosimab treatment and, therefore, 2 sections per brain, from the center of the lesion, as identified in the IBA-1 analysis were selected for this analysis.

#### ChAT and NeuN immunohistochemistry

ChAT-positive fibers and NeuN positive nuclei were measured in layer 5 of the somatosensory cortex. Four (ChAT) or three (NeuN) sections (coordinates respective to Bregma: from − 0.6 to − 1 mm) per brain were analyzed using ICY bioimage analysis software. The sections were selected based on the center of NMDA injections and previously obtained IBA-1 microglia activation relative to bregma. For ChAT analysis, the threshold was determined by applying a gaussian blur (2 × 2 pixel kernel) to reduce noise and a bottom hat line-filter to determine the difference between the input image and the edited image. Following that, the area of ChAT-positive fibers was calculated as the difference in coverage of ChAT active fibers and the total sampling area using the following equation: $\frac{Area \,\,coverage\,\, of\,\, ChAT\,\, or\,\,NeuN\,\,positive\,\,signal }{{total\,\,sampling\,\,area}} \times 100$.

One mouse from group 1) NMDA + FcΔab had to be excluded from the ChAT analysis due to a high background in the immunohistochemical staining as well as many artifacts. Additionally, the number of ChAT-positive cells in the NBM was counted by applying a threshold that distinguishes cells from background, which were counted using the ‘’analyze particles’’ plugin of ImageJ.

For NeuN, the difference in coverage of NeuN positive nuclei and the total sampling area in layer V of the somatosensory cortex was measured. Then, the ratio between the surface area of NeuN positive nuclei in the lesioned and the non-lesioned hemisphere in layer V of the somatosensory cortex was calculated.

#### Synapsin-1 immunohistochemistry

Synapsin-1 coverage was calculated as the difference in coverage of synapsin-1 positive signal and the total sampling area in the hippocampus. Then, the percentage of coverage between the lesioned and non-lesioned (control) hemisphere was calculated.

For all analyses, the images were randomized and visually assessed by an experimenter blinded for experimental conditions.

### Statistics

The data are presented as mean ± SEM. Graphs were generated with Graphpad Prism (version 9.1.2). Normal distribution of the data was analyzed using Shapiro–Wilk normality test. Statistical analyses were conducted using two-sided Student’s t-test as well as one-way ANOVA with post-hoc Tukey test. *p* < 0.05 (*), *p* < 0.01 (**), *p* < 0.001 (***) and *p* < 0.0001 (****) were considered statistically significant.

### Ethics declaration

All procedures were approved by the Animal Welfare Body and Ethical Committee for the use of experimental animals of the University of Groningen, and the Dutch Central Committee on Animal Studies under license number AVD1050020197306.

## Supplementary Information

Supplementary Figures.

## Data Availability

All data generated or analyzed during this study are included in this published article (and its Supplementary Information files).
