# Supplementary material for: The TNFR1 antagonist Atrosimab reduces neuronal loss, glial activation and memory deficits in an acute mouse model of neurodegeneration
Source: Sci Rep. 2023 Jun 30;13:10622. doi: 10.1038/s41598-023-36846-2 (PMC10313728; doi:10.1038/s41598-023-36846-2)
Supplement: Supplementary file 1 — Supplementary Figures. [file 41598_2023_36846_MOESM1_ESM.docx]

**The TNFR1 antagonist Atrosimab reduces neuronal loss, glial activation and memory deficits in an acute mouse model of neurodegeneration**Natalia Ortí-Casañ^a¶*^, Ate S. Boerema^a, b¶^, Karina Köpke^a^, Amber Ebskamp^a^, Jan Keijser^a^, Yuequ Zhang^f^, Tingting Chen^a,f^, Amalia M. Dolga^f^, Kerensa Broersen^c^, Roman Fischer^d, e^, Klaus Pfizenmaier^d, e^, Roland E. Kontermann^d, e^ and Ulrich L. M. Eisel^a*^

^a^ Department of Molecular Neurobiology, Groningen Institute for Evolutionary Life Sciences, University of Groningen, Groningen, The Netherlands

^b^Applied Research Center, Van Hall Larenstein University of Applied Science, Leeuwarden, The Netherlands
^c^ Applied Stem Cell Technology, Faculty of Science and Technology, University of Twente, Enschede, The Netherlands

^d^ Stuttgart Research Center Systems Biology, University of Stuttgart, Stuttgart, Germany

^e^ Institute of Cell biology and Immunology, University of Stuttgart, Stuttgart, Germany

^f^Department of Molecular Pharmacology, Groningen Research Institute of Pharmacy, University of Groningen, The Netherlands

^¶^Authors contributed equally

*Correspondence and material requests should be addressed to N.O-.C ([n.orti.casan@rug.nl](mailto:n.orti.casan@rug.nl)) or U.E ([u.l.m.eisel@rug.nl](mailto:u.l.m.eisel@rug.nl)).

**Supplementary information**


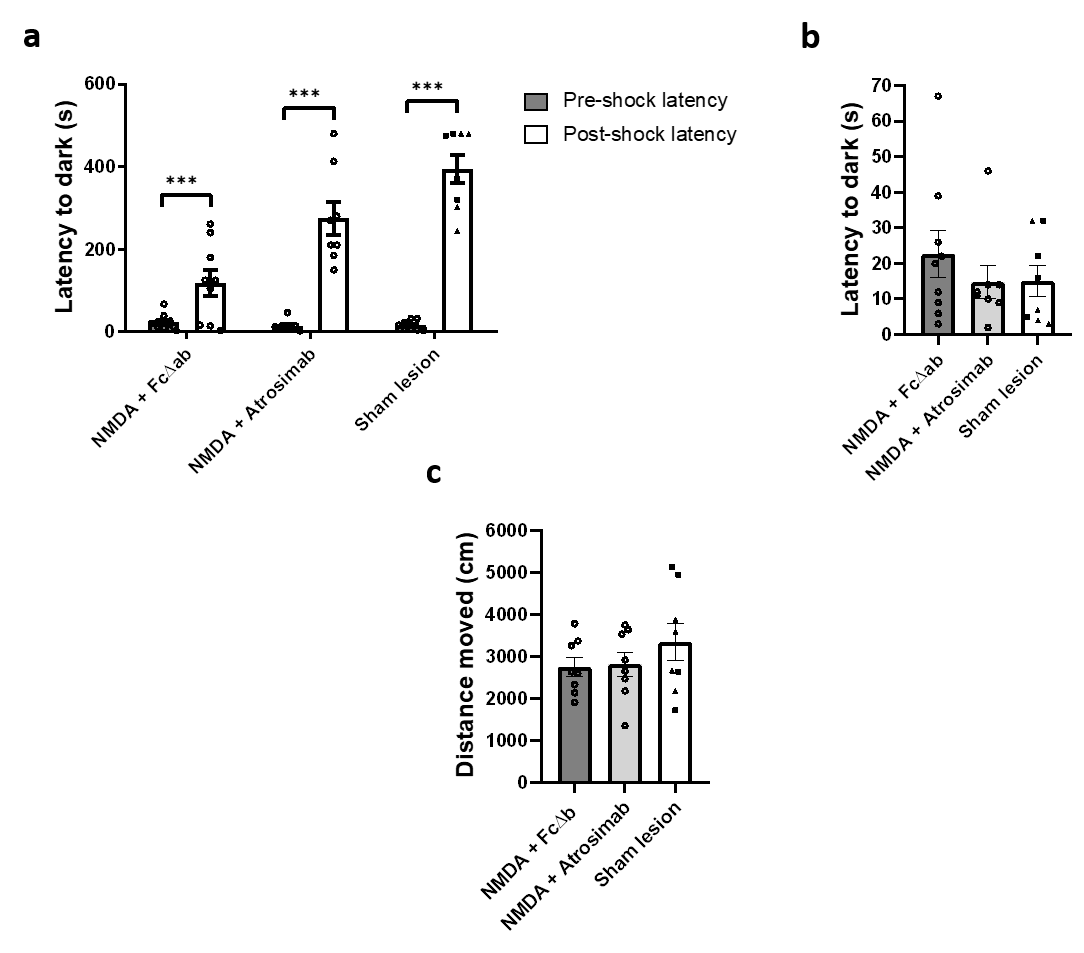


**Supplementary Figure 1**. **Pre-shock latencies and locomotion are not affected after Atrosimab treatment**. **a** Pre- and post-shock latencies were measured in the passive avoidance paradigm. **b** Comparison of pre-shock latencies in the passive avoidance paradigm. **c** Total distanced moved (cm) in the EPM test (NMDA + FcΔab n=9; NMDA + Atrosimab n=8; Sham lesion n=8; one-way ANOVA, Tukey post hoc analysis). The two subgroups combined in the Sham lesion group are represented as follows: ^▲^ represents the Sham + FcΔab subgroup and ^■^ represents the Sham + Atrosimab subgroup, where no significant differences between subgroups were observed. Data are presented as mean ± SEM. ***p<0.001.

**Supplementary Figure 2. Atrosimab does not affect neuronal firing rates.** Primary cortical neurons were pre-treated with Atrosimab and incubated with glutamate. Neuronal firing rates were measured by microelectrode array (Control n=8; Atrosimab n=8; Glutamate n=8; Glutamate + Atrosimab n=8; paired t-tests). Data are presented as mean ± SEM. **p<0.01.


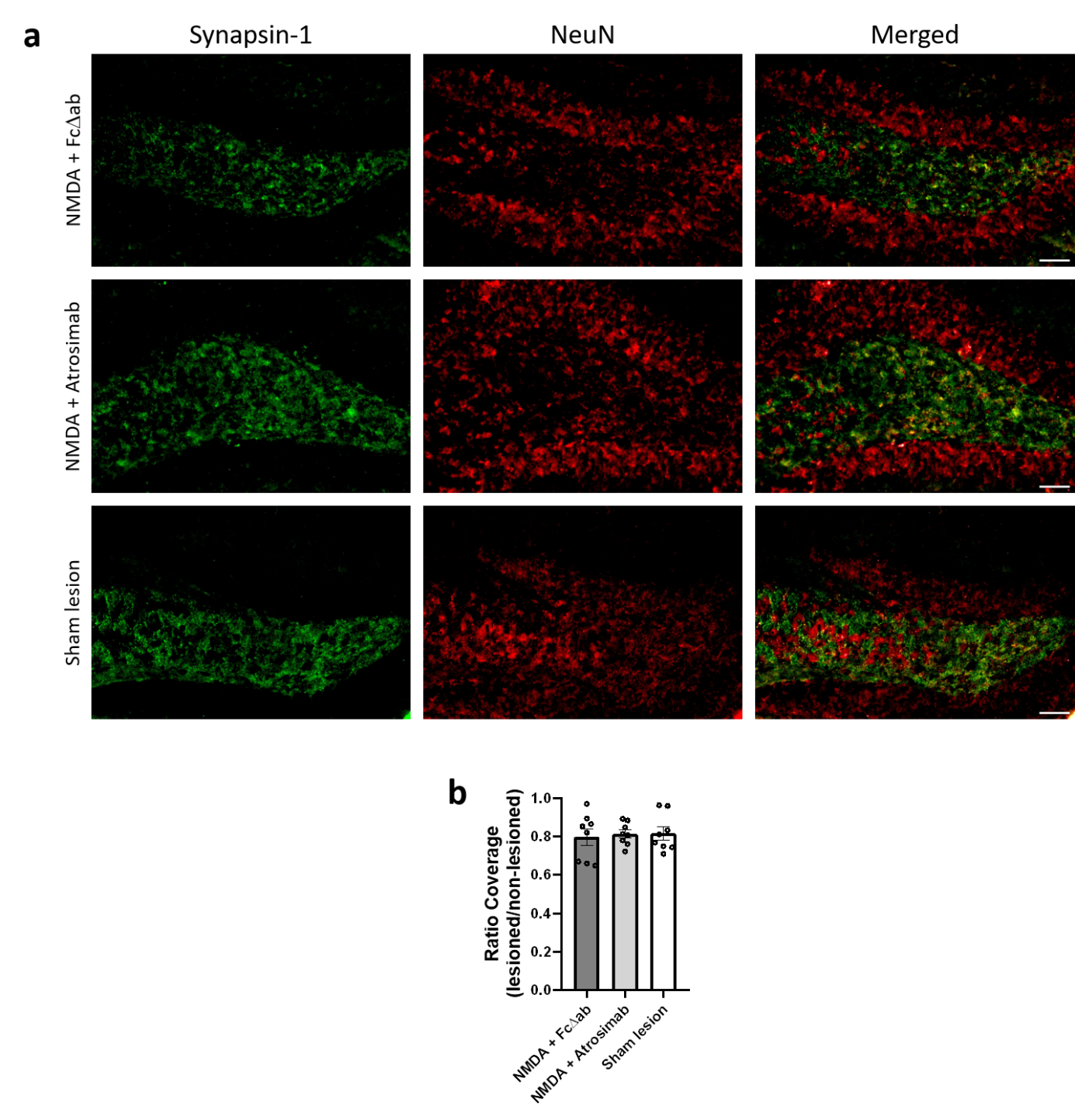


**Supplementary Figure 3. Synapsin-1 levels are not altered by NMDA or Atrosimab co-injections. a** Synapsin-1 (green) and NeuN (red) measured in hippocampal sections. Representative images of hippocampus are shown. Scale bar, 50 µm **b** Quantification of the ratio between the fraction covered by synapsin-1- positive signal in the lesioned vs. non-lesioned hemisphere in **a** (NMDA + FcΔab n=8; NMDA + Atrosimab n=8; Sham lesion n=8; one-way ANOVA, Tukey post hoc analysis). Data are presented as mean ± SEM.
